# Anti-inflammatory and anti-cancer activity of mulberry (*Morus alba* L.) root bark

**DOI:** 10.1186/1472-6882-14-200

**Published:** 2014-06-25

**Authors:** Hyun Ji Eo, Jae Ho Park, Gwang Hun Park, Man Hyo Lee, Jeong Rak Lee, Jin Suk Koo, Jin Boo Jeong

**Affiliations:** 1 Department of Bioresource Sciences, Andong National University, Andong 760749, South Korea; 2 Department of Medicinal Plant Science, Jungwon University, Goesan 367805, South Korea; 3 Gyeongbuk Institute for Bio-industry, Andong 760380, South Korea; 4 Insititute of Agricultural Science and Technology, Andong National University, Andong 760380, South Korea; 5 Department of Medicinal Plant Resources, Andong National University, Andong 760380, South Korea

**Keywords:** *Morus alba* L. Mulberry root bark, Medicinal plant, Anti-inflammation, Anti-cancer

## Abstract

**Background:**

Root bark of mulberry (*Morus alba* L.) has been used in herbal medicine as anti-phlogistic, liver protective, kidney protective, hypotensive, diuretic, anti-cough and analgesic agent. However, the anti-cancer activity and the potential anti-cancer mechanisms of mulberry root bark have not been elucidated. We performed in vitro study to investigate whether mulberry root bark extract (MRBE) shows anti-inflammatory and anti-cancer activity.

**Methods:**

In anti-inflammatory activity, NO was measured using the griess method. iNOS and proteins regulating NF-κB and ERK1/2 signaling were analyzed by Western blot. In anti-cancer activity, cell growth was measured by MTT assay. Cleaved PARP, ATF3 and cyclin D1 were analyzed by Western blot.

**Results:**

In anti-inflammatory effect, MRBE blocked NO production via suppressing iNOS over-expression in LPS-stimulated RAW264.7 cells. In addition, MRBE inhibited NF-κB activation through p65 nuclear translocation via blocking IκB-α degradation and ERK1/2 activation via its hyper-phosphorylation. In anti-cancer activity, MRBE deos-dependently induced cell growth arrest and apoptosis in human colorectal cancer cells, SW480. MRBE treatment to SW480 cells activated ATF3 expression and down-regulated cyclin D1 level. We also observed that MRBE-induced ATF3 expression was dependent on ROS and GSK3β. Moreover, MRBE-induced cyclin D1 down-regulation was mediated from cyclin D1 proteasomal degradation, which was dependent on ROS.

**Conclusions:**

These findings suggest that mulberry root bark exerts anti-inflammatory and anti-cancer activity.

## Background

Inflammation is an innate immune response by various immune cells including macrophages for the protection against the harmful stimuli such as virus and bacteria [1]. As a consequence of excessive inflammatory response, large amounts inflammatory mediators, such as nitric oxide (NO) and prostaglandin E_2_ (PGE_2_) are produced [2]. Inflammatory mediators-induced chronic inflammation is considered to be a cause of numerous human diseases including cancer, atherosclerosis, arthritis and septic shock [3-5]. Among inflammatory mediators, NO is produced by inducible nitric oxide synthase (iNOS) and results in many disease processes such as carcinogenesis, obesity and diabetes [6-8]. iNOS in macrophage is activated following infection. Therefore, iNOS-mediated NO is a ubiquitous mediator of a wide range of inflammatory conditions and reflects degree of inflammation, thus providing a measure of the inflammatory process [9].

It has been reported that development of cancer is associated with inflammation [10,11]. Cancer is a major problem of public health in USA with the estimated 1.6 million new cancer cases and 5.8 hundred thousand cancer deaths occur in USA in 2013 [12]. Among inflammation-induced cancers, colorectal cancer is the third leading cause of cancer-related morbidity and mortality in USA [12]. Recently, cancer chemoprevention has received a great attention and medicinal plants have been regarded as effective anti-cancer sources [13].

Root bark of mulberry (*Morus alba* L.) has been used in herbal medicine as anti-phlogistic, liver protective, kidney protective, hypotensive, diuretic, anti-cough and analgesic agent [14]. However, the anti-cancer activity and the potential anti-cancer mechanisms of mulberry root bark have not been elucidated.

Activating transcription factor 3 (ATF3) is a member of the ATF/CREB subfamily of the basic-region leucine zipper (bZIP) family. In human colorectal cancer, ATF3 expression was suppressed compared to normal adjacent tissue [15]. Up-regulation of ATF3 expression can induce apoptosis in colorectal cancer cells. In addition, ATF3 induces p53 activation [16,17] and inhibits *Ras*-mediated carcinogenesis and cyclin D1 expression [18]. Cyclin D1 regulates cell cycle transition from G1 to S phase by forming cyclin-dependent kinase (CDK) 4 and 6 [19]. Cyclin D1 overexpression was observed in 68.3% of human colorectal cancer [20]. Thus, up-regulation of ATF3 and down-regulation of cyclin D1 are major molecular targets for treatment of colorectal cancer.

In light of the therapeutic potential of mulberry root bark in inflammation-induced colorectal cancer, this study was performed to elucidate the biological mechanism by which mulberry root bark inhibits an inflammatory response in LPS-stimulated RAW264.7 cells and induces the inhibition of cell growth and apoptosis in human colorectal cancer cells. Here, for the first time, we reported that mulberry root bark extracts attenuated NO production by suppressing iNOS expression via regulating the activations of NF-κB and ERK1/2. In addition, it induced cell growth arrest and apoptosis by activating ATF3 expression and cyclin D1 proteasomal degradation in colorectal cancer cells, SW480.

## Methods

### Materials

Cell culture media, Dulbecco’s Modified Eagle medium (DMEM) was purchased from Gibco Inc. (NY, USA). LPS (Escherichia coli 055:B5) and 3-(4,5-dimethylthiazol-2-yl)-2.5-diphenyltetrazolium bromide (MTT) were purchased from Sigma–Aldrich (St. Louis, MO, USA). SB203580, PD98059 were purchased from Calbiochem (San Diego, CA). SB216763 and N-Acetyl Cysteine (NAC) were purchased from Sigma–Aldrich. Antibodies against iNOS, ATF3 and cyclin D1 were purchased from Santa Cruz Biotechnology, Inc (Santa Cruz, CA, USA). Other antibodies against IκB-a, p65, ERK1/2, phospho-ERK1/2 (Thr202/Tyr204) and b-actin were purchased from Cell Signaling (Bervely, MA, USA). All chemicals were purchased from Sigma-Aldrich, unless otherwise specified.

### Sample preparation

The plant sample, Mulberry (*Morus alba* L. voucher number: PARK1002(ANH)) root bark, was kindly provided by the Bonghwa Alpine Medicinal Plant Experiment Station, Korea. One kilogram of mulberry root bark was extracted with 1000 ml of 80% methanol with shaking for 24 h. After 24 h, the methanol-soluble fraction was filtered and concentrated to approximately 20 ml volume using a vacuum evaporator and then fractioned with petroleum ether and ethyl acetate in a separating funnel. The ethyl acetate fraction was separated from the mixture, evaporated by a vacuum evaporator, and prepared aseptically and kept in a refrigerator until use.

### Cell culture and treatment

Mouse macrophage cell line, RAW264.7 and human colorectal cancer cell line, SW480 were purchased from Korean Cell Line Bank (Seoul, Korea) and grown in DMEM supplemented with 10% fetal bovine serum (FBS), 100 U/ml penicillin, and 100 μg/ml streptomycin. These cells were maintained at 37°C under a humidified atmosphere of 5% CO_2_. Mulberry root bark extracts (MRBE) were dissolved in dimethyl sulfoxide (DMSO) and then treated to cells. DMSO was used as a vehicle and the final DMSO concentration was not exceeded 0.1% (v/v).

### Measurement of nitric oxide (NO) production

Inhibitory effect of mulberry root bark extracts on the production of NO in LPS-stimulated RAW264.7 cells was evaluated using literature [21]. Briefly, RAW264.7 cells were plated in 12-well plate for overnight. Cells were pre-treated with mulberry root bark extracts at the indicated concentrations for 2 h and then co-treat with LPS (1 μg/ml) for the additional 18 h. After 18 h, 200 μl of the media was mixed with equal amount of Griess reagent (1% sulfanilamide and 0.1% N-1-(naphthyl) ethylenediamine-diHCl in 2.5% H_3_PO_4_). The mixture was incubated for the additional 5 min at the room temperature and the absorbance was measured at 540 nm.

### Isolation of cytosol and nuclear fraction

Nuclear and cytosolic fractions were prepared following the manufacturer’s protocols of nuclear extract kit (Active Motif, Carlsbad, CA, USA). Briefly, RAW264.7 cells were washed with ice-cold PBS containing phosphatase inhibitors and harvested with 1xhypotonic buffer for 15 min at 4°C. After adding detergent, the cells were centrifuged at 15,000 rpm for30 min and then the supernatants were collected as cytoplasmic fraction. Nuclear fractions were collected by suspending nuclear pellet with lysis buffer and centrifugation.

### MTT assay

The 3-(4,5-dimethylthizaol-2-yl)-2,5-diphenyl tetrazolium bromide (MTT) assay was used to measure cell proliferation. Briefly, SW480 cells were seeded onto 96-well culture plate at a density of 50,000 cells per well. The cells were treated with MRBE for 24 h. Then, 50 μl of MTT solution (1 mg/ml) was added to each well. The resulting crystals were dissolved in DMSO. The formation of formazan was measured by reading absorbance at a wavelength of 570 nm.

### SDS-PAGE and Western blot

Cells were washed with 1 × phosphate-buffered saline (PBS), and lysed in radioimmunoprecipitation assay (RIPA) buffer (Boston Bio Products, Ashland, MA, USA) supplemented with protease inhibitor cocktail (Sigma Aldrich) and phosphatase inhibitor cocktail (Sigma Aldrich), and centrifuged at 12,000 × g for 10 min at 4°C. Protein concentration was determined by the bicinchoninic acid (BCA) protein assay (Pierce, Rockford, IL, USA) using bovine serum albumin (BSA) as the standard. The proteins were separated on SDS-PAGE and transferred to PVDF membrane (Bio-Rad Laboratories, Inc., Hercules, CA, USA). The membranes were blocked for non-specific binding with 5% nonfat dry milk in Tris-buffered saline containing 0.05% Tween 20 (TBS-T) for 1 h at room temperature and then incubated with specific primary antibodies in 5% nonfat dry milk at 4°C overnight. After three washes with TBS-T, the blots were incubated with horse radish peroxidase (HRP)-conjugated immunoglobulin G (IgG) for 1 h at room temperature and chemiluminescence was detected with ECL Western blotting substrate (Amersham Biosciences) and visualized in Polaroid film.

### Reverse transcriptase-polymerase chain reaction (RT-PCR)

Total RNA was prepared using a RNeasy Mini Kit (Qiagen, Valencia, CA, USA) and total RNA (1 μg) was revese-transcribed using a Verso cDNA Kit (Thermo Scientific, Pittsburgh, PA, USA) according to the manufacturer’s protocol for cDNA synthesis. PCR was carried out using PCR Master Mix Kit (Promega, Madison, WI, USA) with primers for human ATF3, human cyclin D1 and human GAPDH as follows: human ATF3: 5′-gtttgaggattttgctaacctgac-3′, and reverse 5′-agctgcaatcttatttctttctcgt-3′ ; human cyclin D1: forward 5′-aactacctggaccgcttcct-3′ and reverse 5′-ccacttgagcttgttcacca-3′ ; huaman GAPDH: forward 5′-acccagaagactgtggatgg-3′ and reverse 5′-ttctagacggcaggtcaggt-3′.

### Statistical analysis

Statistical analysis was performed with the Student’s unpaired t-test, with statistical significance set at *, P < 0.05.

## Results

### The effect of MRBE on NO production and iNOS expression in LPS-stimulated RAW264.7 cells

**Figure 1:**
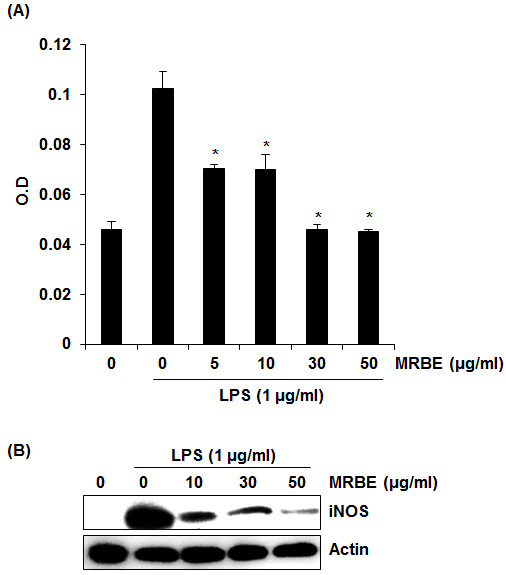
**Effect of MRBE on NO production (A) and iNOS (B) in LPS-stimulated RAW264.7 cells.** RAW264.7 cells were pre-treated with MRBE at the indicated concentrations for 2 h and then co-treated with LPS (1 μg/ml) for the additional 18 h. After treatment, NO production was measured using the media and Griess reagent and cell lysates were resolved by SDS-PAGE, transferred to PVDF membrane, and probed with iNOS antibody for Western blot. iNOS protein was visualized using ECL detection. Actin was used as internal control. DMSO was used as a vehicle. Values given are the mean ± SD (n = 3). *p < 0.05 compared to LPS treatment without MRBE.

Macrophages play an important role in inflammatory response by producing inflammatory mediators such as NO, PGE_2_ and TNF-α [22]. So, we used the mouse macrophage cell line RAW264.7 cells for evaluating anti-inflammatory effect of MRBE. iNOS-mediated NO is associated with cytotoxicity and tissue damage and involved in several processes such as chronic inflammation and immunoregulation [23]. To determine if MRBE could reduce NO generation by LPS, RAW264.7 cells were pretreated with MRBE for 2 h and then co-treated with LPS (1 μg/ml) for the additional 18 h. As shown in Figure 1A, treatment of LPS without MRBE induced NO overproduction in LPS-stimulated RAW264.7 cells, while pretreatment of MRBE suppressed LPS-mediated NO overproduction. Since NO production is regulated by iNOS expression, the effect of MRBE on iNOS expression was evaluated by Western blot. As shown in Figure 1B, LPS overexpression was detected in the cells stimulated LPS alone. However, MRBE inhibited iNOS expression in LPS-stimulated RAW264.7 cells. From these results, MRBE-induced decrease of NO production may result from the inhibition of LPS-induced iNOS overexpression in RAW264.7 cells.

### Inhibitory effect of MRBE on LPS-induced NF-κB and ERK1/2 activation in RAW264.7 cells

**Figure 2:**
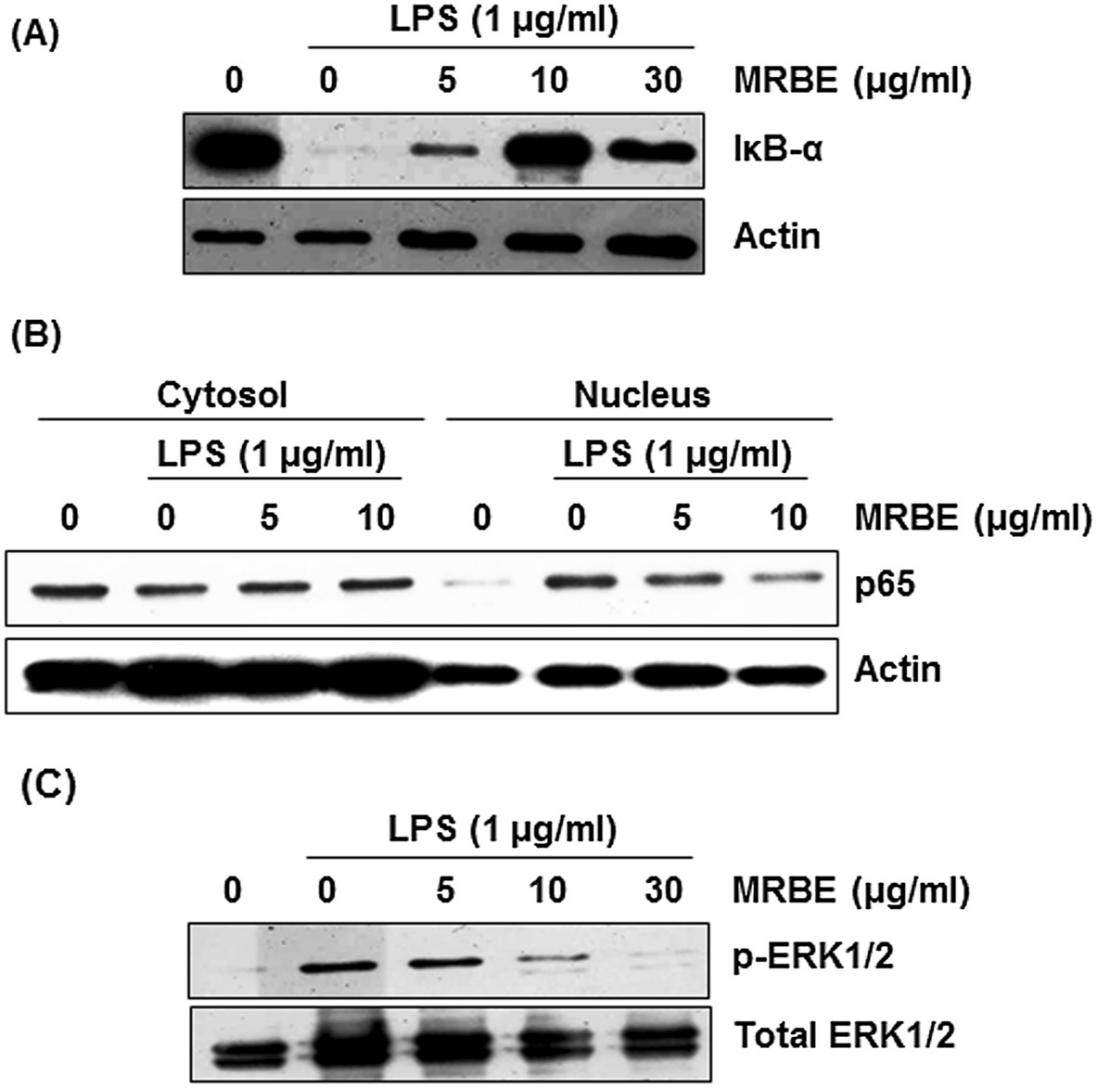
**Effect of MRBE on IκB-α degradation (A), p65 nuclear translocation (B) and ERK1/2 phosphorylation (C) in LPS-stimulated RAW264.7 cells.** RAW264.7 cells were pre-treated with MRBE at the indicated concentrations for 2 h and then co-treated with (1 μg/ml) for 15 min (for Western blot of IκB-α and ERK1/2 phosphorylation) or 30 min (for Western blot of p65). DMSO was used as a vehicle. Cell lysate were resolved by SDS-PAGE, transferred to PVDF membrane, and probed with antibodies against IκB-α, p-ERK1/2, total ERK1/2 and p65. The proteins were then visualized using ECL detection. Actin was used as an internal control.

To elucidate the effect of MRBE on NF-κB activation, we performed a Western blot for IκB-α degradation in LPS-stimulated RAW264.7 cells. As shown in Figure 2A, LPS induced IκB-α degradation at 15 min after the stimulation. However, pretreatment of MRBE attenuated IκB-α degradation in a dose-dependent manner. p65 nuclear translocation resulted from IκB-α degradation are essential in NF-κB activation. Thus we examined whether MRBE inhibits p65 nuclear translocation. As shown in Figure 2B, LPS markedly increased an amount of p65 in the nucleus of RAW264.7 cells. However, pretreatment of MRBE dose-dependently inhibited LPS-induced p65 nuclear translocation in RAW264.7 cells. There is a growing evidence that NF-κB activation is modulated by ERK1/2 activation [24]. Thus, we evaluated the effects of MRBE on phosphorylation of ERK1/2 in LPS-stimulated RAW264.7 cells using Western blot to further investigate whether inhibition of NF-κB activation by MRBE was associated with modulation of ERK1/2. As shown in Figure 2C, Increase of phosphorylation of ERK1/2 was observed in LPS-stimulated RAW264.7 cells without MRBE. However, MRBE attenuated LPS-induced ERK1/2 phosphorylation. Overall, these results suggest that MRBE may inhibit the inflammatory response by ERK1/2-mediated NF-κB activation in LPS-stimulated RAW264.7 cells.

### Effect of MRBE on cell viability and apoptosis in human colorectal cancer cell line, SW480

**Figure 3:**
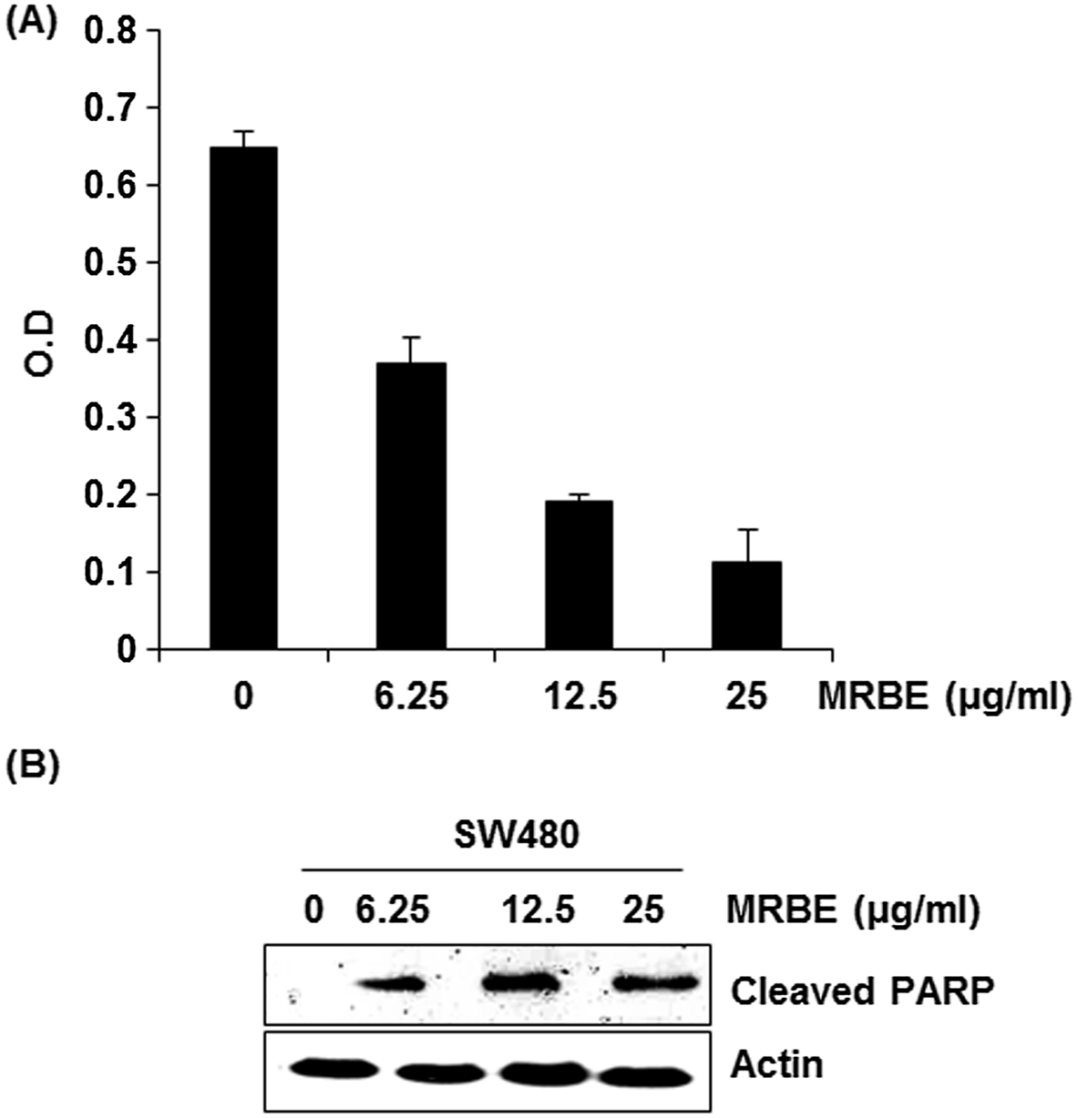
**Effect of MRBE on cell growth (A) and apoptosis (B) in SW480 cells.** SW480 cells were treated with MRBE at the indicated concentration for 24 h. Cell growth was measured sung MTT solution and expressed as absorbance (A_570_). *P < 0.05 compared to cell without MRBE treatment. Apoptosis by MRBE was evaluated with Western blot against cleaved PARP. Actin was used as an internal control. DMSO was used as a vehicle.

There are a number of evidences indicating that MRBE shows anticancer activity in human leukemia cells [25,26]. To observe whether MRBE affects viability and apoptosis in human colorectal cancer cell line, SW480, we carried out MTT assay for cell viability and Western blot for apoptosis. As shown in Figure 3A, treatment of MRBE dose-dependently reduced the viability of SW480 cells by 43%, 71% and 83% at 6.25 μg/ml, 12.5 μg/ml and 25 μg/ml, respectively. Apoptosis was also induced by MRBE treatment (Figure 3B).

### Effect of MRBE on the levels of ATF3 and cyclin D1 in protein and mRNA in SW480 cells

**Figure 4:**
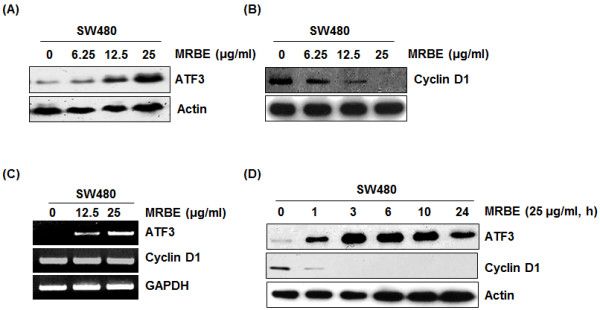
**Effects of MRBE on ATF3 and cyclin D1 expression in mRNA and protein level in SW480 cells. (A, B)** SW480 cells were treated with MRBE at the indicated concentrations for 24 h. Cell lysates were subjected to SDS–PAGE and the Western blot was performed using antibodies against cyclin D1, ATF3 and actin. For RT-PCR analysis of ATF3 and cyclin D1 gene expression **(C)**, total RNA was prepared after MRBE treatment for 24 h. **(D)** SW480 cells were treated with MRBE (25 μg/ml) for the indicated times. Cell lysates were subjected to SDS–PAGE and the Western blot was performed using antibodies against cyclin D1, ATF3 and actin. Actin and GAPDH were used as internal control for Western blot and RT-PCR, respectively. DMSO was used as a vehicle.

There is growing evidence that activating transcription factor 3 (ATF3) is linked to cell growth arrest and apoptosis in colorectal cancer. To investigate whether MRBE activates ATF3 expression in human colorectal cancer cells, SW480 cells were treated with MRBE at the indicated concentrations for 24 h. As shown in Figure 4A and 4C, MRBE induced ATF3 expression in the levels of both protein and mRNA. Time-course experiment showed that induction of ATF3 by MRBE occurred after 1 h stimulation (Figure 4D).We also evaluated whether MRBE regulates cyclin D1 level in SW480 cells since cyclin D1 is associated with cell growth arrest and apoptosis. As shown in Figure 4B, MRBE decreased the protein level of cyclin D1 in a dose-dependent manner. However, decrease in mRNA level of cyclin D1 by MRBE treatment was not observed (Figure 4C). In time-course experiment for cyclin D1 (Figure 4D), MRBE significantly reduced cyclin D1 protein level after 1 h stimulation.

### GSK3β and ROS-dependent ATF3 activation of MRBE in SW480 cells

**Figure 5:**
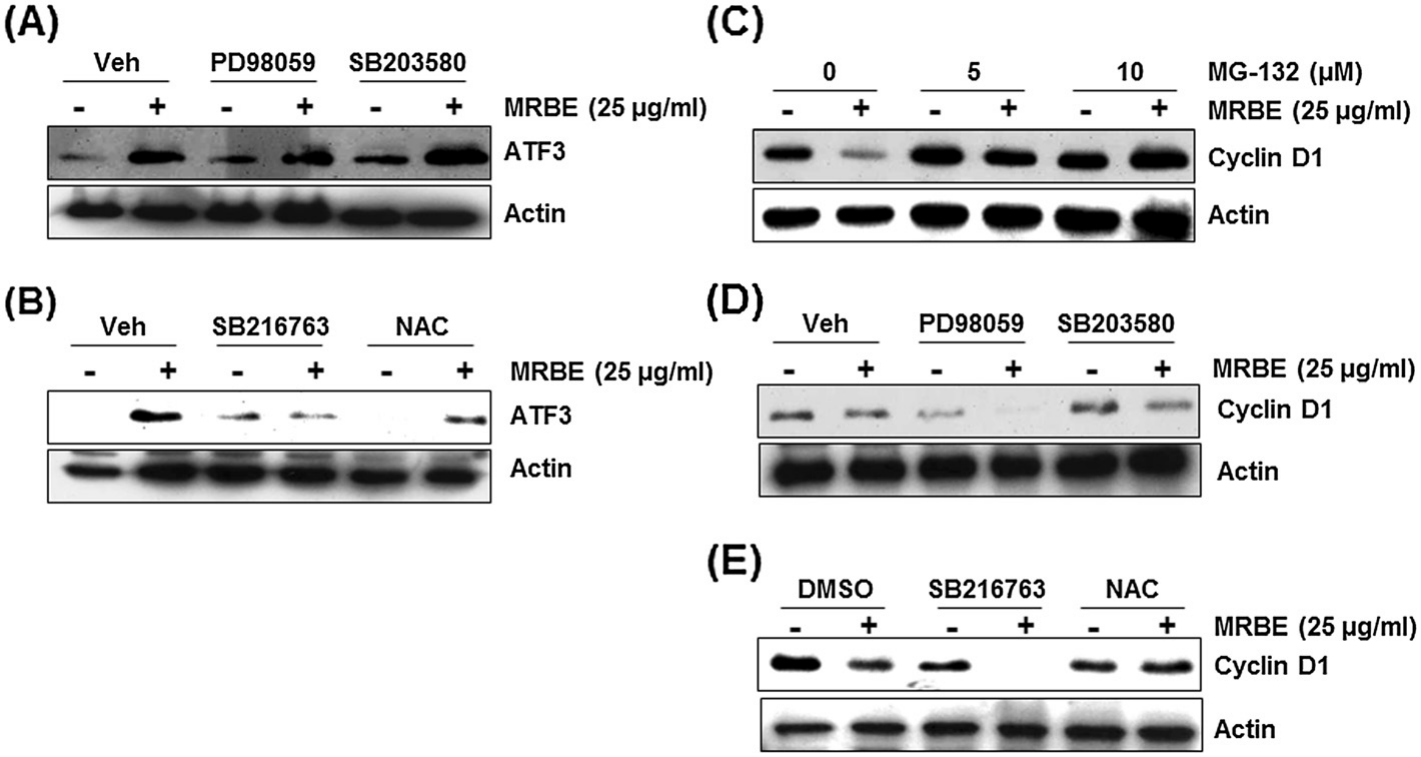
**ROS/GSK3β-dependent ATF3 expression and ROS-dependent cyclin D1 proteasomal degradation by MRBE. (A, B)** SW480 cells were pre-treated with PD98059 (20 μM), SB203580 (20 μM), SB216763 (20 μM) or NAC (20 mM) for 2 h and then co-treated with MRBE (25 μg/ml) for 1 h. Cell lysates were subjected to SDS–PAGE and the Western blot was performed using antibodies against ATF3 and actin. **(C, D, E)** SW480 cells were pre-treated with MG132 (5 and 10 μM), PD98059 (20 μM), SB203580 (20 μM), SB216763 (20 μM) or NAC (20 mM) for 2 h and then co-treated with MRBE (25 μg/ml) for 1 h. Cell lysates were subjected to SDS–PAGE and the Western blot was performed using antibodies against cyclin D1 and actin. Actin was used as an internal control and DMSO was used as a vehicle.

To elucidate the molecular mechanism for MRBE-induced ATF3 expression, we evaluated several signaling pathways affected by MRBE. SW480 cells were pretreated with kinase inhibitors such as PD98059 (ERK1/2 inhibitor), SB203580 (p38 inhibitor) and SB216763 (GSK-3β inhibitor), and NAC (ROS scavenger) for 2 h prior to incubation with 25 μg/ml of MRBE. As shown in Figure 5A, MRBE-induced ATF3 expression was observed in the cells pretreated with PD98059 and SB203580. However, pretreatments of SB216763 and NAC diminished MRBE-induced ATF3 expression (Figure 5B). Collectively, these results suggest the pathways of GSK-3β and ROS may be involved in MRBE-induced ATF3 expression.

### ROS-dependent cyclin D1 proteasomal degradation by MRBE in SW480 cells

We found that MRBE dose-dependently attenuated cyclin D1 protein level whiles it did not affect cyclin D1 mRNA level (Figure 4B and 4C). Thus, we asked whether cyclin D1 protein stability was affected by MRBE treatment. Figure 5C shows that MG-132, a well-known proteasome inhibitor, completely blocked MRBE-induced cyclin D1 down-regulation. This result suggests that MRBE-induced cyclin D1 down-regulation may result from proteasomal degradation. SW480 cells were pretreated with kinase inhibitors such as PD98059, SB203580, SB216763 and NAC for 2 h, and then co-treated with 25 μg/ml of MRBE for the additional 1 h to elucidate the molecular mechanism for cyclin D1 proteasomal degradation affected by MRBE. As shown in Figure 5D and 5E, MRBE-induced cyclin D1 down-regulation was observed in the cells pretreated with PD98059, SB203580 and SB216763. However, NAC attenuated cyclin D1 down-regulation by MRBE. These findings suggest that MRBE induced ROS-dependent cyclin D1 proteasomal degradation.

## Discussion

Although controlled inflammatory response is beneficial to defend and protect the body from harmful factors such as physical damage, precursor chemicals and microbial invasion, inflammation can induce adverse effects such as cancer, atherosclerosis, arthritis and septic shock on the body if the regulation of inflammation is dysfunctional [27]. Since inflammation is associated with many inflammatory mediators such and pathways that lead to a wide range of changes in pathology, it is difficult to treat inflammation [27]. Non-steroidal anti-inflammatory drugs (NSAIDs) have long been used for the treatment of inflammation. However, recently, because of side effects of NSAIDs, herbal medicinal plants have received a great attention and a large number of mechanistic studies have been reported.

Nitric oxide (NO) plays an important role in inflammation pathogenesis [28]. Although NO has an anti-inflammatory effect under controlled inflammatory response, it induces chronic inflammation due to over-production under abnormal regulation [28]. In addition, NO is involved in inflammation-induced human diseases such as cancer, rheumatoid arthritis, diabetes, septic shock and cardiovascular diseases [29,30]. Therefore, NO is a key target for managing inflammatory diseases. NO is synthesized by nitric oxide synthases (NOSs) from the amino acid l-arginine [31,32]. Among three types NOS isoforms including neuronal NOS (nNOS), endothelial NOS (eNOS) and inducible NOS (iNOS), only iNOS is overexpressed in LPS-stimulated macrophage and subsequently generates NO [33]. Mulberry root bark extracts (MRBE) attenuated the over-production of NO by suppressing LPS-induced iNOS over-expression in RAW264.7 cells. These results suggest that MRBE may exert an anti-inflammatory effect.

There are growing evidences that several agents inhibiting NO production have been shown to suppress NF-κB activation [34-37]. NF-κB regulates expression of various genes associated with apoptosis, proliferation, cancer progression and inflammation [38]. In inflammatory response under the external stimuli such as LPS, Activated IκB-α kinase (IKK) phosphorylates IκB-α. Phosphorylated IκB-α is subsequently ubiquitinated and degraded by the 26S proteasome, which thereby releases NF-κB from the cytoplasmic NF-κB-IκBα complex and results in NF-κB nuclear translocation. Translocated NF-κB activates the expressions of target genes associated with inflammation such as iNOS. Thus, NF-κB is the key transcription factor inducing inflammatory response and a promising target for anti-inflammation [39,40]. In present study, we demonstrated that MRBE inhibits the nuclear translocation of NF-κB p65 via blocking LPS-induced IκB-α degradation in RAW264.7 cells.

In addition, some agents inhibiting NO production and NF-κB activation also suppressed the activation of mitogen-activated protein kinases (MAPKs) [37]. The activation of JNK, p38 and ERK1/2 protein regulates iNOS expression and modulates NF-κB activity [41]. We found that MRBE inhibits phosphorylation of ERK1/2 induced by LPS in RAW264.7 cells. These results suggest that inhibition of NF-κB and ERK pathway is a potential mechanism by which MRBE exerts anti-inflammatory activity.

The connection between inflammation and the development of colorectal cancer is well-established [42]. Thus, we evaluated whether MRBE possesses anti-cancer activity and elucidated its potential mechanisms in human colorectal cancer cells, SW480.

ATF3 has been known as a stress-responsive product [43]. There are growing evidences that the ATF3 expression was repressed in normal cells but might be rapidly induced by various pathological stimuli [44,45]. Moreover, ATF3 expression plays an important role in apoptosis induced by a variety of anti-cancer compounds such as berberine [46], conjugated linoleic acid [47], curcumin [48] and 3,3′-diindolylmethane [49] in human colorectal cancer cells, which indicates that ATF3 could function as a pro-apoptotic mediator. We found that MRBE induced cell growth arrest and apoptosis, and activated ATF3 expression in the levels of mRNA and protein in SW480. We also found that MRBE-activated ATF3 expression was reduced by the treatments SB216763 (GSK3β inhibitor) and NAC (ROS scavenger), which indicates that MRBE-induced ATF3 expression could be dependent on GSK3β and ROS. Indeed, there is a report that ROS induced ATF3-mediated apoptosis in human colorectal cancer cells [50]. However, the effect of GSK3β on ATF3 expression has not been elucidated. Thus, it is necessary that MRBE-induced ATF3 is affected by GSK3β directly or indirectly. These results suggest that MRBE could induce apoptosis through GSK3β and ROS-dependent ATF3 activation.

Moreover, we observed that MRBE treatment down-regulated cyclin D1 protein level but not mRNA in SW480 cells. Thus, we hypothesized that MRBE-induced decrease in cyclin D1 level may be mediated from its proteasomal degradation, and found that pretreatment of MG132 (proteasome inhibitor) suppresses MRBE-induced cyclin D1 down-regulation. There are some kinases reported to degrade cyclin D1 such as p38 [51], ERK1/2 [52], GSK3β [53] and ROS [54]. From using kinase inhibitor, we conclude that MRBE-induced cyclin D1 degradation requires ROS; however, p38, ERK1/2 and GSK3β are not involved in MRBE-induced cyclin D1 degradation. Because cyclin D1 is a well-known cell cycle regulatory protein, MRBE-induced cell growth arrest could be mediated from cyclin D1 proteasomal degradation.

Mulberry root bark has been reported to have various active components such as mulberroside A, oxyresveratrol, mulberrofuran G, kuwanon C, kuwanon G, kuwanon H and morusin. Among these active components, kuwanon C and kuwanon G possess an anti-inflammatory effect [55,56]. In anti-cancer activity, morusin induce apoptosis and suppress NF-kB in human colorectal cancer cells [57]. From these studies, anti-inflammatory and anti-cancer activity by MRBE may be contributed by kuwanon C, kuwanon G or morusin. But, we do not exclude the possibility that other active components could also mediate an anti-inflammatory and anti-cancer activity.

## Conclusions

Taken together, our report is the first to show that MRBE exerts anti-inflammatory and anti-cancer activity. Anti-inflammatory effect of MRBE is mediated from inhibiting NF-κB and ERK1/2 activation. Anti-cancer activity of MRBE is associated with ROS-dependent cyclin D1 proteasomal degradation and ROS/ GSK3β-dependent ATF3 expression.

## Abbreviations

MRBE: Mulberry root bark extract; NO: Nitric oxide; iNOS: Inducible nitric oxide synthease; NF-κB: Nuclear factor-kappaB; ERK1/2: Extracellular signal-related kinase 1/2; ATF3: Activating transcription factor 3; ROS: Reactive oxygen species.

## Competing interest

The authors declare that they have no conflict interest.

## Authors’ contributions

JBJ directed and HJE designed the study. HJE, JHP, GHP, MHL, JRL and JSK performed the experiments. HJE and JHP drafted manuscript. JHP, GHP, MHL, JRL, JSK and JBJ corrected the manuscript. All authors read and approved the final manuscript.

## Pre-publication history

The pre-publication history for this paper can be accessed here:

http://www.biomedcentral.com/1472-6882/14/200/prepub
